# (1*R*,1′*S*)-1,1′-Dihydr­oxy-1,1′-biisobenzofuran-3,3′(1*H*,1′*H*)-dione

**DOI:** 10.1107/S1600536809044961

**Published:** 2009-10-31

**Authors:** Fang-Fang Jian, Shan-Shan Zhao, Huan-Mei Guo, Yu-Feng Li, Pu-Su Zhao

**Affiliations:** a Microscale Science Institute, Weifang University, Weifang 261061, People’s Republic of China; b New Materials and Function Coordination Chemistry Laboratory, Qingdao University of Science and Technology, Qingdao 266042, People’s Republic of China

## Abstract

In the title compound, C_16_H_10_O_6_, the complete mol­ecule is generated by a crystallographic centre of symmetry. In the crystal, O—H⋯O hydrogen bonds link the mol­ecules into (100) sheets and C—H⋯O links also occur.

## Related literature

For background to phthalides as natural products, see: Pedrosa *et al.* (2006 ▶). For a related structure, see: Wang *et al.* (2001 ▶). 
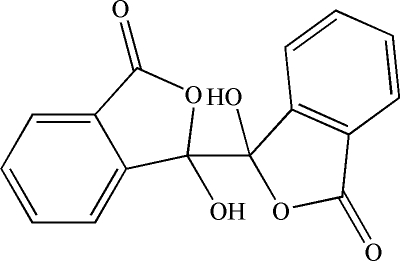


## Experimental

### 

#### Crystal data

C_16_H_10_O_6_
*M* *_r_* = 298.24
Monoclinic, 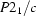

*a* = 8.2260 (16) Å
*b* = 7.9690 (16) Å
*c* = 10.859 (4) Å
β = 114.03 (2)°
*V* = 650.1 (3) Å^3^
*Z* = 2
Mo *K*α radiation
μ = 0.12 mm^−1^
*T* = 293 K
0.16 × 0.12 × 0.10 mm

#### Data collection

Bruker SMART CCD diffractometer
Absorption correction: none
1352 measured reflections
1263 independent reflections
622 reflections with *I* > 2σ(*I*)
*R*_int_ = 0.070

#### Refinement

*R*[*F* ^2^ > 2σ(*F* ^2^)] = 0.056
*wR*(*F* ^2^) = 0.170
*S* = 1.02
1263 reflections
121 parameters
H atoms treated by a mixture of independent and constrained refinement
Δρ_max_ = 0.29 e Å^−3^
Δρ_min_ = −0.30 e Å^−3^

### 

Data collection: *SMART* (Bruker, 1997 ▶); cell refinement: *SAINT* (Bruker, 1997 ▶); data reduction: *SAINT*; program(s) used to solve structure: *SHELXS97* (Sheldrick, 2008 ▶); program(s) used to refine structure: *SHELXL97* (Sheldrick, 2008 ▶); molecular graphics: *SHELXTL* (Sheldrick, 2008 ▶); software used to prepare material for publication: *SHELXTL*.

## Supplementary Material

Crystal structure: contains datablocks I, global. DOI: 10.1107/S1600536809044961/hb5170sup1.cif

Structure factors: contains datablocks I. DOI: 10.1107/S1600536809044961/hb5170Isup2.hkl

Additional supplementary materials:  crystallographic information; 3D view; checkCIF report

## Figures and Tables

**Table 1:** Hydrogen-bond geometry (Å, °)

*D*—H⋯*A*	*D*—H	H⋯*A*	*D*⋯*A*	*D*—H⋯*A*
O2—H2*B*⋯O1^i^	0.91 (7)	1.82 (7)	2.691 (5)	159 (5)
C5—H5*A*⋯O1^ii^	0.96 (3)	2.58 (4)	3.475 (5)	155 (3)

Symmetry codes: (i) 
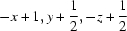
; (ii) 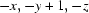.

**Fig. 1.**
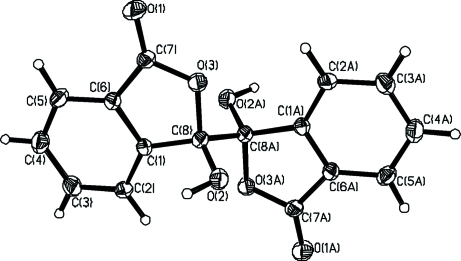
The molecular structure of (I) with displacement ellipsoids drawn at the 30% probability level.

## supplementary crystallographic information

### Comment

Substituted phthalides (isobenzofuran-1(3*H*)-ones) represent an important class of natural products that posses significant biological properties (e.g. Pedrosa *et al.*, 2006). As part of our search for new biologically active compounds, we unexpected obtained the title compound, (I), which is a typical derivative of phthalides.

In the crystal structure of compound (I) (Fig. 1),there is an inversion center, which is located at the mid-point of C(8)—C(8 A) bond. All of the bond lengths and bond angles are in the normal ranges (Wang *et al.*, 2001). In the crystal lattice, there are a C—H···O intramolecular hydrogen bond and an O—H···O intermolecular hydrogen bond, which stabilize the molecule structure.

### Experimental

Phthalic anhydride (0.05 mol) was dissolved in dichloromethane (100 ml). Then, AlCl_3_ (0.05 mol) was added. The mixture was stirred at room temperature and the whole reaction was under the protection of nitrogen. After 5 h, the reaction was stopped and the mixture poured into ice-water. The organic layer was collected and then was dried with MgSO_4_. Finally, the organic layer was concentrated by rotary vacuum evaporation to obtain yellow solids. Yellow blocks of (I) were obtailed by recrystallization from acetonitrile at room temperature.

### Refinement

The H atoms were located in difference maps and freely refined.

### Crystal data

**Table:**

C_16_H_10_O_6_	*F*(000) = 308
*M**_r_* = 298.24	*D*_x_ = 1.523 Mg m^−^^3^
Monoclinic, *P*2_1_/*c*	Mo *K*α radiation, λ = 0.71073 Å
Hall symbol: -P 2ybc	Cell parameters from 1978 reflections
*a* = 8.2260 (16) Å	θ = 3.5–27.5°
*b* = 7.9690 (16) Å	µ = 0.12 mm^−^^1^
*c* = 10.859 (4) Å	*T* = 293 K
β = 114.03 (2)°	Block, yellow
*V* = 650.1 (3) Å^3^	0.16 × 0.12 × 0.10 mm
*Z* = 2	

### Data collection

**Table:**

Bruker SMART CCD diffractometer	622 reflections with *I* > 2σ(*I*)
Radiation source: fine-focus sealed tube	*R*_int_ = 0.070
graphite	θ_max_ = 25.9°, θ_min_ = 2.7°
ω scans	*h* = 0→9
1352 measured reflections	*k* = −9→0
1263 independent reflections	*l* = −13→12

### Refinement

**Table:**

Refinement on *F*^2^	Secondary atom site location: difference Fourier map
Least-squares matrix: full	Hydrogen site location: inferred from neighbouring sites
*R*[*F*^2^ > 2σ(*F*^2^)] = 0.056	H atoms treated by a mixture of independent and constrained refinement
*wR*(*F*^2^) = 0.170	*w* = 1/[σ^2^(*F*_o_^2^) + (0.0846*P*)^2^] where *P* = (*F*_o_^2^ + 2*F*_c_^2^)/3
*S* = 1.02	(Δ/σ)_max_ < 0.001
1263 reflections	Δρ_max_ = 0.29 e Å^−^^3^
121 parameters	Δρ_min_ = −0.30 e Å^−^^3^
0 restraints	Extinction correction: *SHELXL97* (Sheldrick, 2008), Fc^*^=kFc[1+0.001xFc^2^λ^3^/sin(2θ)]^-1/4^
Primary atom site location: structure-invariant direct methods	Extinction coefficient: 0.032 (10)

### Special details

**Table:**

Geometry. All e.s.d.'s (except the e.s.d. in the dihedral angle between two l.s. planes) are estimated using the full covariance matrix. The cell e.s.d.'s are taken into account individually in the estimation of e.s.d.'s in distances, angles and torsion angles; correlations between e.s.d.'s in cell parameters are only used when they are defined by crystal symmetry. An approximate (isotropic) treatment of cell e.s.d.'s is used for estimating e.s.d.'s involving l.s. planes.
Refinement. Refinement of *F*^2^ against ALL reflections. The weighted *R*-factor *wR* and goodness of fit *S* are based on *F*^2^, conventional *R*-factors *R* are based on *F*, with *F* set to zero for negative *F*^2^. The threshold expression of *F*^2^ > σ(*F*^2^) is used only for calculating *R*-factors(gt) *etc*. and is not relevant to the choice of reflections for refinement. *R*-factors based on *F*^2^ are statistically about twice as large as those based on *F*, and *R*- factors based on ALL data will be even larger.

### Fractional atomic coordinates and isotropic or equivalent isotropic displacement parameters (Å^2^)

**Table:**

	*x*	*y*	*z*	*U*_iso_*/*U*_eq_	
O1	0.2464 (4)	0.3665 (4)	0.1300 (3)	0.0549 (9)	
O2	0.6822 (3)	0.5869 (4)	0.4778 (3)	0.0410 (8)	
O3	0.4600 (3)	0.4127 (3)	0.3342 (2)	0.0376 (8)	
C1	0.3733 (5)	0.6782 (5)	0.3741 (3)	0.0314 (9)	
C2	0.3529 (6)	0.8340 (5)	0.4222 (4)	0.0398 (11)	
C3	0.2204 (6)	0.9369 (6)	0.3366 (4)	0.0467 (11)	
C4	0.1095 (6)	0.8864 (6)	0.2078 (4)	0.0484 (12)	
C5	0.1271 (5)	0.7290 (6)	0.1605 (4)	0.0408 (11)	
C6	0.2612 (5)	0.6276 (5)	0.2465 (3)	0.0316 (9)	
C7	0.3138 (5)	0.4584 (5)	0.2258 (3)	0.0359 (10)	
C8	0.5080 (5)	0.5441 (5)	0.4390 (3)	0.0330 (10)	
H2A	0.435 (5)	0.868 (4)	0.511 (4)	0.036 (10)*	
H4A	0.014 (5)	0.956 (6)	0.148 (4)	0.052 (12)*	
H5A	0.052 (5)	0.686 (5)	0.073 (3)	0.036 (10)*	
H3A	0.206 (6)	1.043 (7)	0.368 (4)	0.069 (15)*	
H2B	0.689 (9)	0.669 (8)	0.422 (6)	0.12 (2)*	

### Atomic displacement parameters (Å^2^)

**Table:**

	*U*^11^	*U*^22^	*U*^33^	*U*^12^	*U*^13^	*U*^23^
O1	0.067 (2)	0.049 (2)	0.0364 (15)	0.0066 (16)	0.0091 (15)	−0.0153 (15)
O2	0.0378 (16)	0.0411 (18)	0.0391 (15)	−0.0008 (14)	0.0105 (13)	0.0080 (13)
O3	0.0498 (17)	0.0327 (16)	0.0273 (13)	0.0054 (13)	0.0125 (13)	−0.0038 (12)
C1	0.036 (2)	0.028 (2)	0.0265 (18)	−0.0023 (16)	0.0096 (17)	0.0042 (16)
C2	0.052 (3)	0.032 (2)	0.0280 (19)	−0.003 (2)	0.008 (2)	−0.0055 (18)
C3	0.057 (3)	0.035 (3)	0.046 (2)	0.010 (2)	0.018 (2)	0.000 (2)
C4	0.045 (3)	0.049 (3)	0.043 (2)	0.011 (2)	0.009 (2)	0.008 (2)
C5	0.039 (2)	0.048 (3)	0.028 (2)	0.000 (2)	0.0058 (18)	−0.003 (2)
C6	0.035 (2)	0.032 (2)	0.0263 (18)	0.0009 (17)	0.0107 (17)	0.0015 (16)
C7	0.044 (2)	0.037 (3)	0.0251 (19)	−0.0039 (19)	0.0119 (18)	−0.0032 (17)
C8	0.037 (2)	0.030 (2)	0.0262 (18)	0.0016 (18)	0.0074 (17)	−0.0005 (16)

### Geometric parameters (Å, °)

**Table:**

O1—C7	1.207 (4)	C2—H2A	0.96 (4)
O2—C8	1.362 (4)	C3—C4	1.382 (6)
O2—H2B	0.91 (7)	C3—H3A	0.94 (5)
O3—C7	1.346 (4)	C4—C5	1.385 (6)
O3—C8	1.477 (4)	C4—H4A	0.96 (4)
C1—C6	1.376 (5)	C5—C6	1.380 (5)
C1—C2	1.383 (5)	C5—H5A	0.96 (4)
C1—C8	1.494 (5)	C6—C7	1.461 (5)
C2—C3	1.378 (6)	C8—C8^i^	1.551 (7)
			
C8—O2—H2B	108 (4)	C6—C5—H5A	118 (2)
C7—O3—C8	110.2 (3)	C4—C5—H5A	125 (2)
C6—C1—C2	120.6 (4)	C1—C6—C5	122.2 (4)
C6—C1—C8	109.2 (3)	C1—C6—C7	107.9 (3)
C2—C1—C8	130.2 (3)	C5—C6—C7	129.9 (3)
C3—C2—C1	117.6 (4)	O1—C7—O3	121.5 (4)
C3—C2—H2A	123 (2)	O1—C7—C6	129.2 (4)
C1—C2—H2A	119 (2)	O3—C7—C6	109.3 (3)
C2—C3—C4	121.6 (5)	O2—C8—O3	109.5 (3)
C2—C3—H3A	118 (3)	O2—C8—C1	116.8 (3)
C4—C3—H3A	120 (3)	O3—C8—C1	103.2 (3)
C3—C4—C5	120.9 (4)	O2—C8—C8^i^	107.1 (4)
C3—C4—H4A	122 (2)	O3—C8—C8^i^	104.4 (4)
C5—C4—H4A	117 (2)	C1—C8—C8^i^	115.0 (4)
C6—C5—C4	117.1 (4)		

Symmetry codes: (i) −*x*+1, −*y*+1, −*z*+1.

### Hydrogen-bond geometry (Å, °)

**Table:**

*D*—H···*A*	*D*—H	H···*A*	*D*···*A*	*D*—H···*A*
O2—H2B···O1^ii^	0.91 (7)	1.82 (7)	2.691 (5)	159 (5)
C5—H5A···O1^iii^	0.96 (3)	2.58 (4)	3.475 (5)	155 (3)

Symmetry codes: (ii) −*x*+1, *y*+1/2, −*z*+1/2; (iii) −*x*, −*y*+1, −*z*.
